# Deep learning-driven incidental detection of vertebral fractures in cancer patients: advancing diagnostic precision and clinical management

**DOI:** 10.1007/s11547-025-02058-z

**Published:** 2025-08-02

**Authors:** El Mehdi Mniai, Vladimir Laletin, Lambros Tselikas, Tarek Assi, Baptiste Bonnet, Astrid Orfali Camez, Amir Zemmouri, Serge Muller, Tania Moussa, Yasmina Chaibi, Julie Kiewsky, Sarah Quenet, Christophe Avare, Nathalie Lassau, Corinne Balleyguier, Angela Ayobi, Samy Ammari

**Affiliations:** 1 Department of Radiology, Gustave Roussy, 94805 Villejuif, France; 2 Avicenna.AI, 375 Avenue du Mistral, 13600 La Ciotat, France; 3 Département d’Anesthésie, Chirurgie et Interventionnel (DACI), Service de Radiologie Interventionnelle, Gustave Roussy, 94805 Villejuif, France; 4 Centre d’Investigation Clinique BIOTHERIS, INSERM CIC 1428, 94805 Villejuif, France; 5 Laboratoire d’Imagerie Biomédicale Multimodale Paris-Saclay, Inserm, CNRS, CEA, BIOMAPS, UMR 1281, Université Paris-Saclay, 94800 Villejuif, France; 6 Division of International Patients Care, Gustave Roussy, 94805 Villejuif, France; 7 Mohammed VI University of Sciences and Health – UM6SS, Casablanca, Morocco

**Keywords:** Vertebral compression fracture, VCF, Deep-learning, Cancer, AI, Radiology

## Abstract

**Purpose:**

Vertebral compression fractures (VCFs) are the most prevalent skeletal manifestations of osteoporosis in cancer patients. Yet, they are frequently missed or not reported in routine clinical radiology, adversely impacting patient outcomes and quality of life. This study evaluates the diagnostic performance of a deep-learning (DL)-based application and its potential to reduce the miss rate of incidental VCFs in a high-risk cancer population.

**Materials and methods:**

We retrospectively analysed thoraco-abdomino-pelvic (TAP) CT scans from 1556 patients with stage IV cancer collected consecutively over a 4-month period (September–December 2023) in a tertiary cancer center. A DL-based application flagged cases positive for VCFs, which were subsequently reviewed by two expert radiologists for validation. Additionally, grade 3 fractures identified by the application were independently assessed by two expert interventional radiologists to determine their eligibility for vertebroplasty.

**Results:**

Of the 1556 cases, 501 were flagged as positive for VCF by the application, with 436 confirmed as true positives by expert review, yielding a positive predictive value (PPV) of 87%. Common causes of false positives included sclerotic vertebral metastases, scoliosis, and vertebrae misidentification. Notably, 83.5% (364/436) of true positive VCFs were absent from radiology reports, indicating a substantial non-report rate in routine practice. Ten grade 3 fractures were overlooked or not reported by radiologists. Among them, 9 were deemed suitable for vertebroplasty by expert interventional radiologists.

**Conclusion:**

This study underscores the potential of DL-based applications to improve the detection of VCFs. The analyzed tool can assist radiologists in detecting more incidental vertebral fractures in adult cancer patients, optimising timely treatment and reducing associated morbidity and economic burden. Moreover, it might enhance patient access to interventional treatments such as vertebroplasty. These findings highlight the transformative role that DL can play in optimising clinical management and outcomes for osteoporosis-related VCFs in cancer patients.

**Supplementary Information:**

The online version contains supplementary material available at 10.1007/s11547-025-02058-z.

## Introduction

Vertebral fractures are the most common type of fracture linked to osteoporosis, accounting for up to 50% of all fractures in individuals with this condition [1–3]. These fractures predominantly involve vertebral compression fractures (VCFs) of the thoracic and lumbar vertebral bodies [4, 5]. The overall prevalence of vertebral fractures worldwide was estimated at approximately 20% [6]. Despite this high prevalence, VCFs frequently remain asymptomatic and clinically undetected, resulting in underdiagnosis and suboptimal patient management [7–9]. Undiagnosed VCFs are associated with increased mortality, a heightened risk of subsequent fractures—particularly hip neck fractures—and a greater likelihood of immobility [1, 10–12]. Collectively, these factors contribute to poor prognoses and significantly diminished quality of life [13, 14].

Cancer patients face an elevated risk of vertebral fractures. Indeed, the spine is the most frequent site of skeletal metastases, which trigger osteoclast activation, irregular bone trabeculae formation, and bone resorption [15, 16]. Cancer therapies like chemotherapy agents and glucocorticoid treatment can reduce bone mineral mass through direct bone toxicities and indirect sex hormone lowering mechanisms [17, 18]. Additionally, sex hormone level lowering is a strategy for the treatment of hormone-dependent tumors [17, 19]. Moreover, cancer predominantly affects an aging population, which is already susceptible to bone pathology and osteoporosis due to age-related declines in sex hormone levels [19]. Radiotherapy and radionuclide treatments, as well as cancer immunotherapy, favor bone mineral loss and bone fractures [17, 20–22]. Hence, testing for VCF and osteoporosis might significantly improve cancer patient management and outcomes.

Computer tomography (CT) is a widespread examination in cancer patients and was demonstrated to be of high usability for opportunistic osteoporosis and VCF fracture screening [23, 24]. However, the implementation of opportunistic pathology detection may be slowed by the excessive demand placed on radiologists. With the advancement of deep learning (DL) solutions in radiology, this task can be at least partially automated, reducing the clinical workload and enhancing radiologists’ efficiency [25, 26]. Deep learning applications in radiology are undergoing significant advancements, demonstrating their potential as complementary tools to augment radiologists’ performance, reduce involvement in repetitive tasks, and mitigate professional fatigue [27–29]. Multiple tools for automated osteoporosis and VCF screening have been proposed recently [30–40]. In this study, we aimed to assess the diagnostic performance of an automated deep learning tool for VCF detection and quantification (CINA-VCF Quantix, Avicenna.AI, La Ciotat, France) in an asymptomatic oncology population undergoing CT imaging as part of their regular oncological care. Furthermore, we examined the potential impact on clinical management identifying patients with VCF that would benefit from vertebroplasty and that were not reported by radiologists during standard evaluations.

## Materials and methods

### Ethical considerations

This study was approved by the Institutional Review Board of Gustave Roussy Cancer Campus (no.: 2024-381). The need for written informed consent was waived. Avicenna.AI provided the VCF detection DL-based algorithm for this study. The study received no financial support.

### Data collection and study design

All consecutive thoraco-abdomino-pelvic (TAP) CT scans performed from September to December 2023 at the Institute Gustave Roussy (Villejuif, France) for patients with stage IV cancer were retrospectively collected. CT scans were acquired on GE Healthcare GE Optima CT660 (GE Healthcare, Milwaukee, WI, USA) and Siemens Healthineers SOMATOM® Force Dual Source CT system (Siemens Healthineers, Erlangen, Germany). Demographic data of included patients were collected, including age, gender, cancer type, bone metastatic status, and spinal treatment status.

All collected cases were analyzed by CINA-VCF Quantix DL-based application version 0.7 (Avicenna.AI, La Ciotat, France) for VCF detection. The cases flagged positive for VCF (grade 1, grade 2, and grade 3, corresponding to—20–25%, 26–40%, and > 40% of vertebral height loss, respectively, according to Genants’ classification [41]) were reviewed by two expert radiologists (one senior and one junior) in order to validate the VCF findings. The DL-based application performance was retrospectively compared to the radiological assessment, based on the available reports.

All grade 3 fractures detected by DL application were independently analyzed by two expert interventional radiologists, with 10 and 2 years of experience respectively, to assess whether they would have treated these severe vertebral fractures with vertebroplasty or not.

Finally, as previously reported, to evaluate osteoporosis risk, anterior trabecular CT attenuation values of the L1 vertebra in mean Hounsfield units (HU) were evaluated [42]. Using a region-of-interest (ROI) approach, mean HU values were automatically computed by the DL application for all scans in which the L1 vertebra was visible in the absence of sclerotic metastases and fractures.

### Deep learning algorithm and training methodology

The DL-based algorithm, developed by Avicenna.AI (CINA-VCF Quantix v0.7, La Ciotat, France), was developed and trained as previously reported [43]. In brief, the algorithm was built on 2D/3D U-Net-based CNN architectures. It identifies and standardizes the spine, detects and labels thoracic and lumbar vertebrae, and excludes vertebrae with cement or other materials. The model was trained on 12,402 vertebrae collected from 886 cases sourced from both U.S. and French centers between 2021 and 2022. The datasets encompassed a broad clinical diversity, ensuring balanced representation across scanner manufacturers, patient age groups, genders, contrast types, fields of view, and slice thicknesses. Specifically, the vertebral height loss (VHL) algorithm was trained on a representative subset of the training dataset comprising 325 cases, representing 3576 individually annotated vertebrae. Each vertebral level between T1 and L5 was represented, with L1 the lowest frequency (100 occurrences) and T11–T12 the highest (198 occurrences), reflecting the targeted fields of view coverage. The annotation process was the same for the training and pilot validation datasets, resulting for each vertebra in six coplanar points per vertebra corresponding to the endpoints of the vertebral body’s anterior, middle, and posterior measurement segments. The model was then trained to reproduce the landmarks positioned on a 2D patch centered around the vertebral body in the sagittal plane defined by the measures. The algorithm quantifies intra and intervertebral height loss relative to neighboring vertebrae, calculate VHL and derives the Genant grade from it. As a final step, an ad-hoc algorithm defines an elliptical region-of-interest (ROI) placement on the mid-vertebra body level, in the axial plane, for mean HU measurement. Outputs include vertebral labels, VHL grades (Genant’s 1–3 grade fractures), and mean HU values for L1–L4 or T8–T11 if the lumbar spine is not in the field of view.

To minimize the biases in the model training, the selection process specifically targeted a distribution of the grades at the vertebra level close to the general population prevalence (89.1% grade 0, 4.5% grade 1, 4.7% grade 2, and 1.7% grade 3). It included 55.7% of patients with at least a vertebra of grade > 0 and 44.3% of patients with at least one confounding factor. Due to the limited number of patients with bone metastasis, data augmentation included Hounsfield Units shifts. A stratified sampling strategy was used during the minibatch construction to compensate for the generally low prevalence of high grades.

The algorithm was evaluated on an independent pilot dataset comprising 1,994 vertebrae from 152 cases. It demonstrated a sensitivity of 92% (95% CI 82–97%), a specificity of 99% (95% CI 93–100%), and an overall accuracy of 96% (95% CI 92–99%) for detecting VCFs. Vertebral labeling reached an accuracy of 98% (95% CI 94.3–99.6%), with a 95% limit of agreement for the height loss at the vertebra level of [− 9.89%, 10.57%] and a bias of 0.34%. A strong correlation (ranging from 0.6 to 0.8) for mean Hounsfield Unit (HU) measurements was achieved.

### Statistical analysis

Per-patient and per-vertebra positive predictive values (PPV) for VCF detection were calculated by comparing DL-based application results with the evaluations made by two clinical expert radiologists who evaluated only flagged as positive by DL application cases. The per-patient and per-vertebra VCF non-reported rates were evaluated by comparing the software results with clinical reports. Finally, the vertebroplasty missed rate was calculated based on the comparison of a documented clinical report and the independent evaluation of two expert interventional radiologists. All the statistical analyses were performed using MedCalc Statistical Software (v20.015, MedCalc Software Ltd., Ostend, Belgium).

## Results

### Study population

**Fig. 1:**
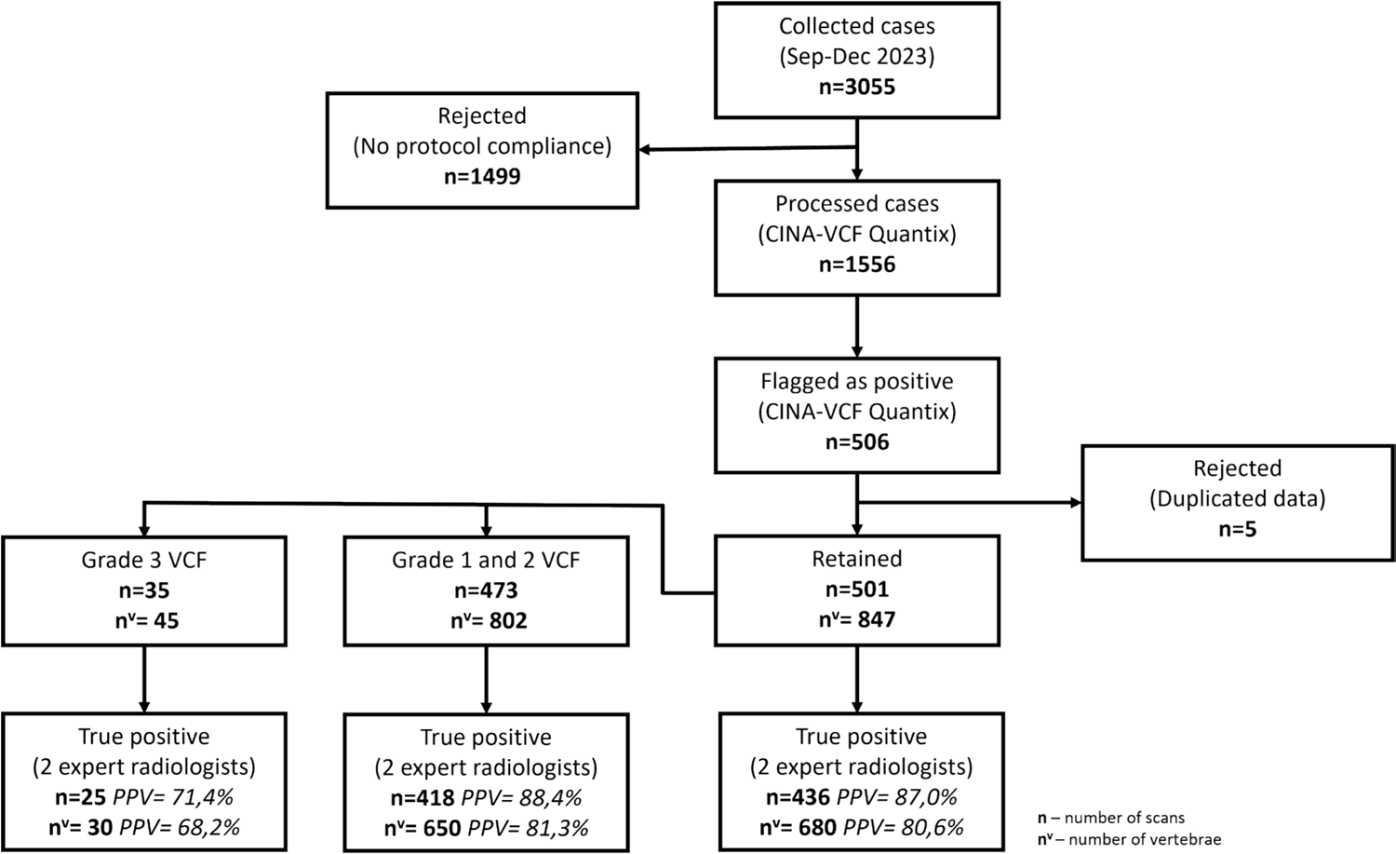
Study flowchart and software performances. From 3,055 collected CT scans, 1556 scans passed the acquisition protocol of the CINA-VCF Quantix application. 501 individual patients were flagged positive and reviewed by two expert radiologists. The number of true positive cases was established and the positive predictive value (PPV) was calculated. The data was analyzed *per scan* and *per vertebrae* level. The PPV was calculated for all fractures and for grade 1–2 and grade 3 fractures separately. Some cases included both grade 1–2 and grade 3 fractures

For this study, a total of 3055 computed tomography (CT) scans were collected over a 4-month period, spanning from September to December 2023. All cases were processed by CINA-VCF Quantix application. Of these, 1499 scans (49.1%) were excluded by the DL-based application due to non-compliance with the acquisition protocol (Fig. 1). The exclusions were attributed to patient age (as the application processes cases from patients aged 50 years and older) and incompatible slice thickness or reconstruction kernel. Among the 1556 CT scans retained in the study, 506 instances were flagged as positive for vertebral compression fracture by the application. After a detailed review, among positively flagged CT scans, five corresponded to secondary examination of the same patients with VCF during the study period, so they were excluded as duplicated data (Fig. 1). Finally, the 501 flagged as positive individual patients by CINA-VCF Quantix were retained for statistical analysis. The mean age for the 501 patients was 63.6 ± 11.5 years, and there were 231 (46.1%) of women. For the patients for whom the data on metastasis, osteoporosis, or interventional treatment of VCF were accessible, the distribution was as follows: mean bone density was 126.04 ± 46.32 HU (*n* = 431); bone metastases were present in 140 (31.46%) out of 445 patients with available data; osteoporosis was present in 217 (50.46%) out of 430 patients with accessible record. Finally, 32 (7.26%) out of 441 patients with available information had previous interventional treatment of VCF. Table 1 and Fig. 1. Supplementary Fig. 1 presents particular situations in the dataset.

**Table 1:** Patient cohort characteristics

Characteristic	Flagged positive by CINA (VCF Quantix) Total: 501	All TP cases Total: 436	Grades 1 and 2 TP fractures Total: 418	Grade 3 TP fractures Total: 25
Mean bone density (L1) *mean HU* ± *SD*	126.04 ± 46.32 (*n* = 431)*	126.00 ± 46.03 (*n* = 421)	127.56 ± 45.61 (*n* = 404)	84.88 ± 40.59 (*n* = 24)
Sex *women, %*	46.10% (231/501)	48.17% (210/436)	47.61% (199/418)	52% (13/25)
Age *mean* ± *SD*	63.59 ± 11.49 (*n* = 501)	63.35 ± 11.56 (*n* = 436)	63.19 ± 11.61 (*n* = 418)	68.36 ± 9.57 (*n* = 25)
Bone metastases *% of patients*	31.46% (140/445)	31.87% (138/433)	31.33% (131/415)	48% (12/25)
Osteoporosis *% of patients*	50.46% (217/430)	50.48% (212/420)	49.63% (200/403)	75% (18/24)
Vertebroplasty *% of patients*	7.26% (32/441)	7.18% (31/433)	6.3% (26/414)	20% (5/25)

*In parenthesis (): number of available data

### Diagnostic performance of the DL-based application

**Fig. 2:**
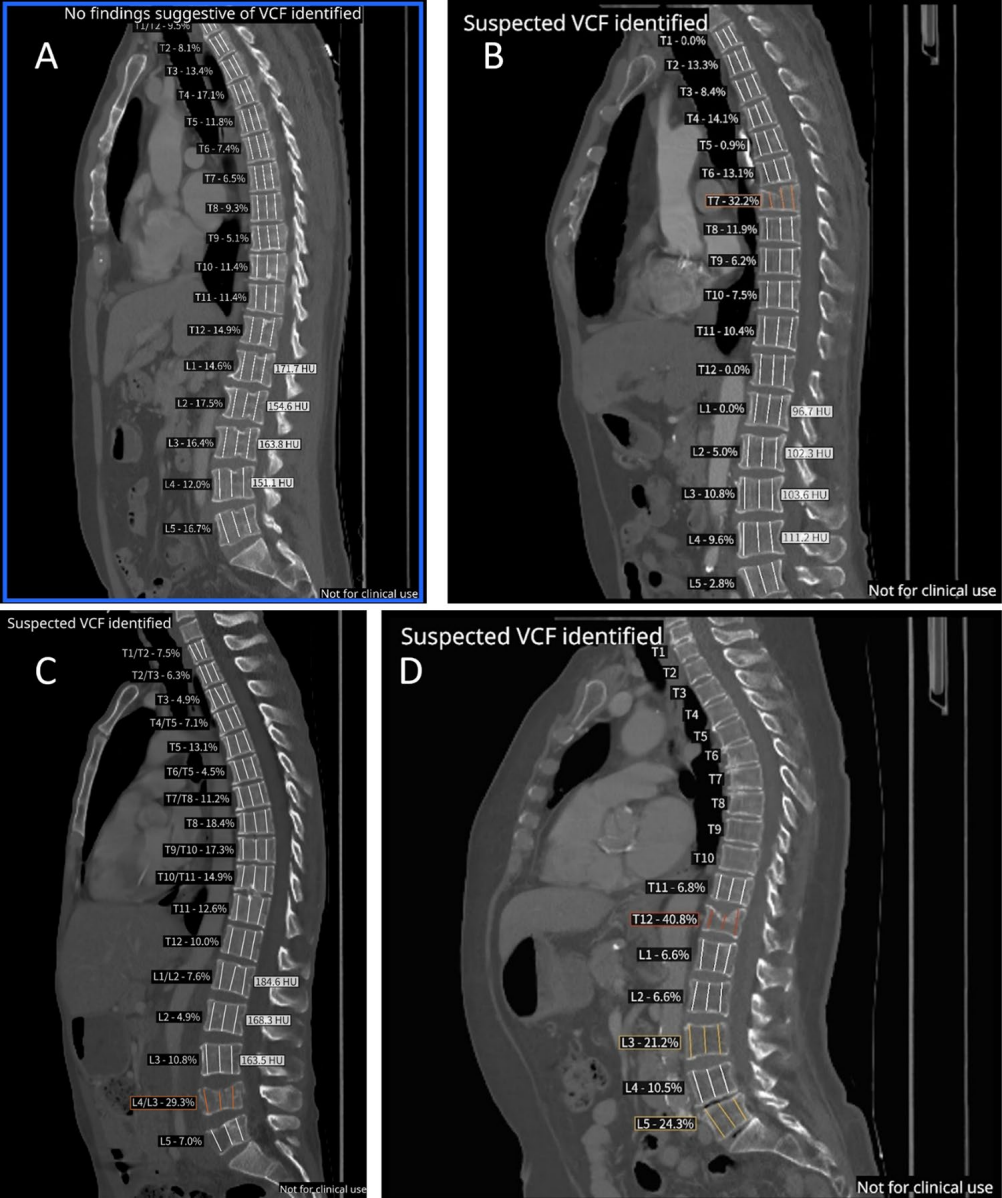
Examples of negative and positive cases detections. **A** Negative case. The DL-based application quantifies intra and intervertebral height loss relative to neighboring vertebrae. The threshold for positive cases is set to 20% of height loss. Variation of height loss is presented in percentage for each vertebra. Mean HU is indicated for L1–L4 vertebrae. **B** Positive case. Grade 2 vertebral fracture of T7 in an osteoporotic patient—Height loss estimated at 32.2%—Mean HU L1 trabecular attenuation: 96.7 HU (physiological reference 122–198 HU). **C** Positive case. Grade 2 vertebral fracture of L4 in a patient with lytic bone metastasis. **D** Positive case. T12 grade 3 vertebral fracture and L3, L5 grade 1 vertebral fracture identified

After a careful review of 501 flagged as positive by DL-based application cases, two expert radiologists in a consensus analysis confirmed 436 cases as true positive (TP) for VCF. Regarding primary tumor distribution for TP cases, lung, head and neck, and skin (excluding melanoma) tumors contributed to 19.2%, 18.8%, and 10% of cases, respectively. Hormone-dependent tumors were equally presented in the dataset with 8.6% of cases with prostate cancer and 7.6% of cases with breast tumors. Supplementary Fig. 2 presents additional data distribution in terms of primary tumor sites. Sixty-five cases were considered as being false positive (FP), leading to a PPV of 87.0% (95% CI 83.8–89.8%) (Fig. 1). Representative examples of true negative (TN) and true positive (TP) notifications are presented in Fig. 2.

**Fig. 3:**
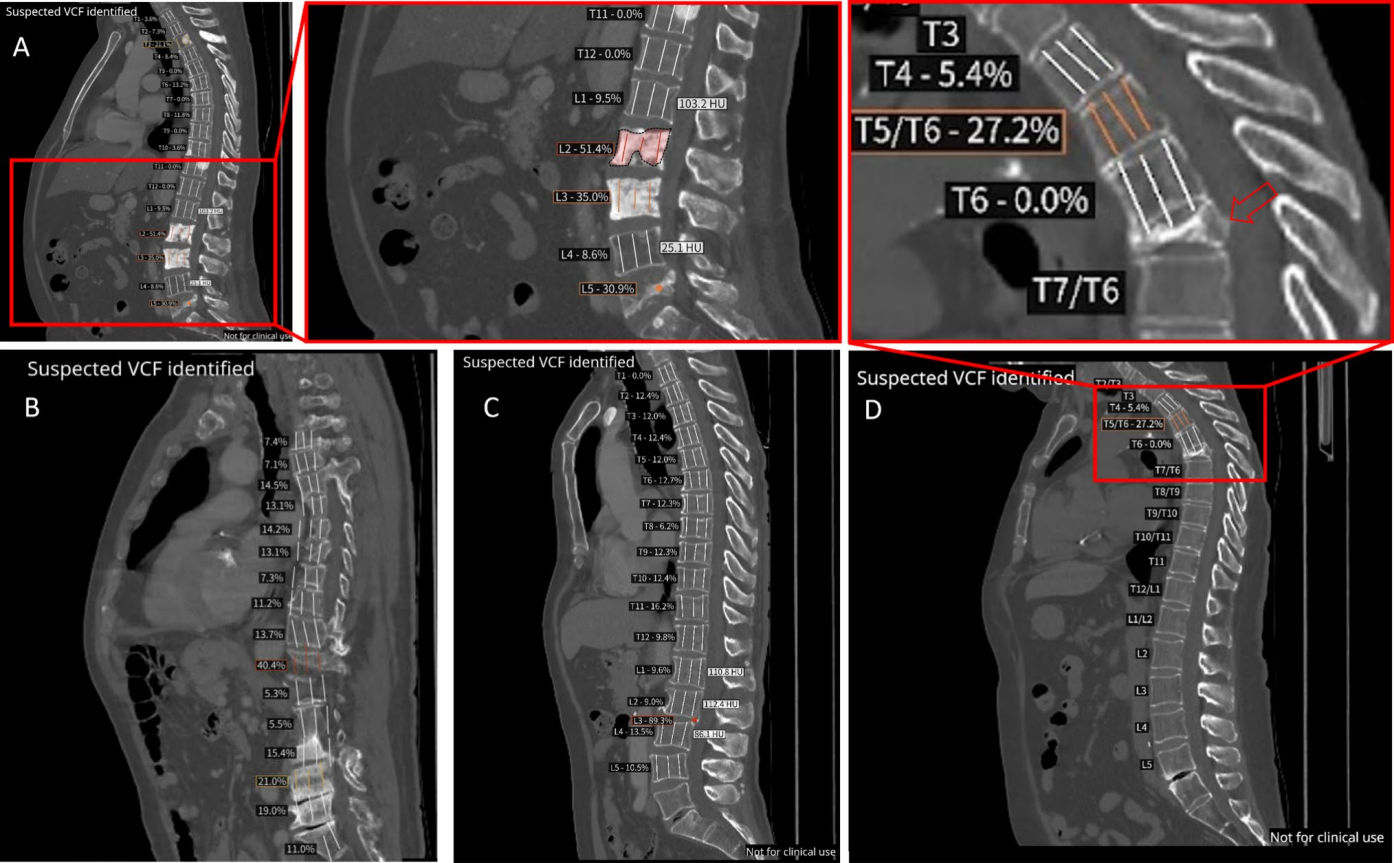
Examples of false positive and false negative notifications. **A** False-positive case due to an error in vertebral height measurement because of sclerotic vertebral metastases. **B** False-positive case due to incorrect height measurement linked to scoliosis. **C** False-positive case due to incorrect vertebra centroid detection. AI displays a circle in the region to alert that the height loss may not be correct. **D** False-negative for grade 3 T7 vertebral fracture

Regarding individual vertebrae, among the 436 true positive cases, there were 683 individual VCFs. Of these, 680 were correctly identified by the DL-based application; hence, three VCFs were missed. Additionally, the application identified 164 vertebrae as fractured; however, expert radiologists classified them as false positives, leading to a per-vertebra PPV of 80.6% (95% CI 77.7–83.2%) (Fig. 1). Examples of false negative and false positive detections are presented in Fig. 3. Regarding the VCF grade, 350 cases were considered as grade 1 (1 FN, 75 FPs, and 274 TPs), 452 cases as grade 2 (1 FN, 75 FPs, and 376 TPs), and 45 cases as grade 3 (1 FN, 14 FPs, and 30 TPs), according to the experts.

The main reasons related to false positives were: sclerotic vertebral metastases, which impede vertebral height measurement; scoliosis; and incorrect centroid detection due to the vertebrae deformation or collapsing (Fig. 3A–C). In this latter case, the DL-based application displays a dot in the region to alert the user that there is a high probability that the displayed height loss may not be correct (Fig. 3C).

Concerning the false negatives **(**Fig. 3D), two main sources of inaccuracies of the current algorithm were identified, related to vertebral body count and vertebral centroid identification. Both sources of false negatives will be addressed in the Discussion.

### Detection of VCFs not reported by radiologists

**Fig. 4:**
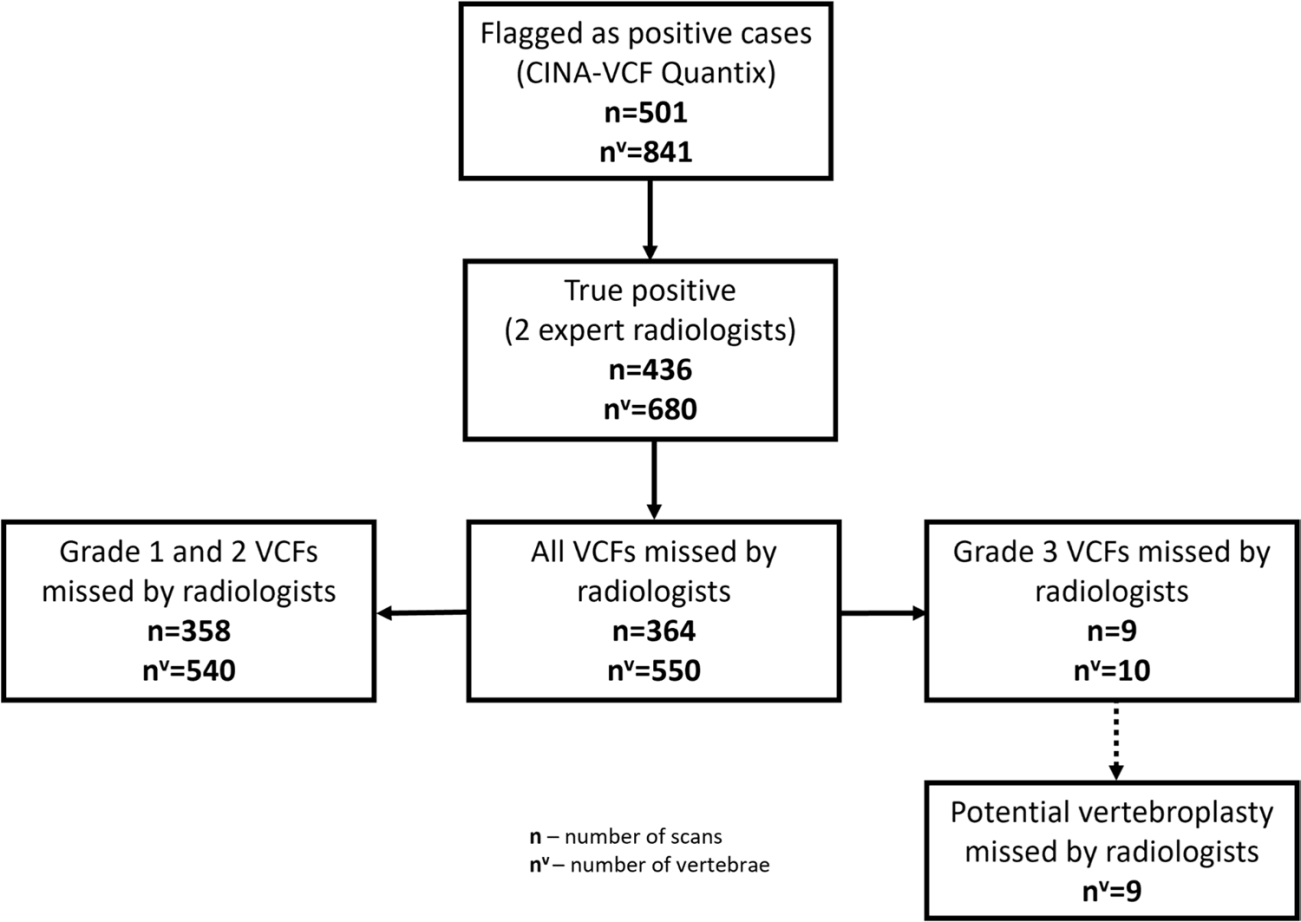
Vertebral fractures not reported by radiologists. Among 501 cases (841 vertebrae) flagged for vertebral fracture by DL-based application, 436 cases (680 vertebrae) were confirmed to be true positives by two expert radiologists. The analysis of radiology reports revealed the number of non-reported fractures by radiologists in general practice. Two expert interventional radiologists independently reviewed severe (grade 3) vertebral fractures. Among 10 non-reported cases by radiologists, nine would have been indicated to vertebroplasty

Among 436 true positive cases, 364 (83.5%) were not mentioned in radiology reports. Therefore, these patients can be considered missed or unreported by radiologists in routine clinical practice (Fig. 4). Regarding per-vertebra findings, of the 680 fractures correctly detected by the DL-based application, 550 (80.9%) were not reported by radiologists (Fig. 4). Of these fractures, 540 were classified as grade 1 or grade 2, accounting for 83% of all 650 true positive grade 1 and grade 2 fractures. These corresponded to 85.7% (358 of 418) of patients. Moreover, 10 grade 3 fractures out of 30 (33.3%) were not reported by radiologists (Fig. 4). These corresponded to 36.0% (9 of 25) of patients.

Regarding the fracture localization, among 650 grade 1 and 2 fractures, 317 (48.8%) thoracic and 315 (48.5%) lumbar vertebrae were fractured. No difference in application performance, neither for thoracic nor for lumbar vertebrae, has been noticed. Among 202 vertebral fractures of patients with grade 1 and 2 fractures presenting vertebral metastases, 41 (20.3%) fractures were related to the metastatic process. Moreover, regarding grade 3 VCFs, 22 (73.3%) out of 30 fractures were localized in the thoracic and 8 (26.6%) in the lumbar spine. Among the 13 fractures of patients with grade 3 VCFs presenting vertebral metastases, 6 (46.2%) fractures were related to the metastatic process.

### Outcomes for clinical management

Finally, two interventional radiology experts, with 10 and 2 years of clinical experience, independently reviewed all grade 3 fractures to assess the potential indication for vertebroplasty in these severe cases. Among the 30 fractures reviewed, cementoplasty was recommended in 27 cases. Of the 10 fractures initially overlooked by radiologists, cementoplasty was deemed appropriate in nine cases (Fig. 4). This analysis highlights that 33% (9/27) of fractures requiring intervention were detected by the DL-based application but were not reported during routine clinical practice by radiologists.

## Discussion

The purpose of this study was to evaluate the diagnostic performance of a DL-based application for incidental VCF detection on routine CT scans of asymptomatic oncological patients. The DL-based tool demonstrated a per-case PPV of 87.0% and a per-vertebra PPV of 80.6% for all VCFs. For grade 3 VCFs, the per-case and per-vertebra PPV were 71.4% and 68.2%. Regarding grade 1–2 VCFs, these values were 88.4% and 81.3%, respectively. Radiologists did not describe in the reports 83.5% of cases with VCF and 80.9% of individual vertebrae fractures detected by DL-based application. Finally, of the 10 vertebrae with grade 3 fractures that were not reported by radiologists and detected by the DL-based tool, nine required an interventional procedure, as assessed by two experienced interventional radiologists.

The automated detection of VCFs is a growing area of research. Various solutions have been proposed across different imaging modalities. Deep learning (DL) tools for standard radiographs have demonstrated high accuracy in VCF detection, exceeding 98.5% [44, 45]. Moreover, these tools have proven to be cost-effective for healthcare systems [46]. Automated VCF detection on MRI has shown performance comparable to that of spine surgeons [47]. Notably, a DL application for MRI-based VCF detection significantly improved accuracy among less-experienced radiologists [48]. DL algorithms for CT-based VCF detection are also advancing, with tools capable of vertebral labeling, segmentation, and metastasis detection [35, 39]. Additionally, CT has proven to be an optimal modality for automated osteoporosis screening, which is particularly relevant for applications detecting VCFs [34, 36, 37]. The performance of DL tools for VCF detection on CT has ranged from 47.4 to 98.7% in sensitivity and from 63.9 to 95.8% in specificity [31, 33, 38, 40]. The application currently under evaluation, CINA-VCF Quantix, previously demonstrated a sensitivity of 92.3% and specificity of 91.7% in a previous study [43].

Regarding the current study, the application demonstrated relatively high PPV values (87.0% for overall per-case PPV). The lowest PPV was documented for grade 3 VCFs (71.4% for overall per-case PPV). It represented 10 false positive cases out of 35 alerts generated by DL-based applications. The main reasons for these false positive detections were sclerotic vertebral metastases, scoliosis, and incorrect centroid detection. All these causes lead to incorrect vertebral height measurement. Given this PPV, radiologists would need to review an additional one-eighth of the flagged cases. However, since these cases are associated with confounding factors and pathologies highly impacting patient life quality, reviewing these cases can be very advantageous. Regarding the previously published literature, PPV of 46.7% [32] and 13.9% [40] have been previously reported for other automated DL-based tools for VCF detection. Therefore, the currently evaluated application performed better and demonstrated interesting performance for its implementation in clinical practice.

Regarding false positives, the DL-based application was found to generate erroneous alerts in the presence of confounding factors such as sclerotic metastases and scoliosis. To mitigate these inaccuracies, a dedicated dataset enriched with cases exhibiting these conditions could be employed for training. However, false positives associated with scoliosis, particularly those arising from inaccurate centroid estimations, might persist despite this strategy. To address this limitation, the implementation of a specialized algorithm for the reorientation of scoliotic vertebrae along the craniocaudal axis might be proposed.

Concerning the false negatives, two main sources of inaccuracies were identified. The first source of inaccuracies takes place at the measure level. The landmark prediction algorithm precision depends both on the acquisition resolution and vertebral body presentation. Because the model somehow learned to identify patterns associated with the vertebral body contours, it can be confused by either anatomical variations or confounding factors that change the general shape or intensity of these contours leading to errors in the height loss ratio and potentially a vertebra classified as grade 0 instead of 1 or more. The second source of inaccuracies takes place in the preliminary steps of general processing where the vertebral body centers are located using a dedicated deep learning model. These locations are associated with a probability for each candidate vertebra. When this probability is below a confidence level, the candidate vertebra is ignored both for the measures and labelling. When the ignored vertebra is the only one with a high grade, the case can be flagged as negative. Nonetheless, future versions of the device will need to address the current limitations revealed by the study. Both sources of errors will clearly benefit from more data, with a focus on adding patients with more high-grade vertebra and confounding factors which are currently either ignored during pre-processing or subject to landmark misplacements. However, given that in the more extreme cases the landmark approach may fail (there is a floor in the landmark location accuracy due to the resolution), we may complement the current approach with a classifier dedicated to flag these extreme conditions independently of the measures themselves.

No difference was observed in the DL application performance for detecting VCF in the thoracic or lumbar spine. This consistency is likely due to the training methodology, in which the model was trained at the single-vertebra level. Vertebral height is assessed individually and compared to neighboring vertebrae to compute the vertebral height loss (VHL) based on intervertebral height differences. As a result, the algorithm operates independently of the anatomical region, exhibiting no bias toward thoracic or lumbar vertebrae.

In this study on an asymptomatic oncology patient population, we reported that among true positive cases detected by DL-based application, radiologists did not report 83.5% of VCF-positive patients. This data aligns with previously published data, suggesting that radiologists do not report 84% of patients with VCFs during routine CT scan examinations [49]. In a previously published study on a restricted cohort of patients (105 cases), 12 (63.2%) out of 19 true positive cases detected by the DL-based tool were not reported by radiologists [32]. This highlights a significant proportion of patients who are overlooked by specific prophylactic measures, as previously reported data indicate that 19.2% of individuals with incidental vertebral fractures are likely to develop new vertebral fractures within a year [50]. Moreover, these osteoporotic patients are at an increased risk of hip fractures, which, alongside vertebral fractures, account for the highest economic burden among all fracture types in public health [51]. Therefore, early detection of this high-risk patient group could prove to be a cost-effective strategy [5, 52, 53]. Among the grade 3 fractures not reported by the radiologist, nine would have been eligible for interventional treatment. Importantly, timely vertebroplasty can reduce pain and lower the risk of subsequent fractures [54]. Therefore, these patients could have experienced substantial clinical benefits if the DL-based tool for VCF detection had been implemented during their examination.

This study highlights that 83.5% of VCFs are not reported by radiologists in standard clinical practice in a tertiary cancer center. However, these results should be reviewed in the prism of a real-world clinical practice addressing several limitations. First, the scans are performed in asymptomatic patients with no clinical indication of VCF, thus the radiologists performed an oncological assessment rather than systematically evaluate VCFs. Factors such as work fatigue and high workload may impact radiologists’ assessments, limiting the time available for thorough case review and reporting, leading to the omission of incidental findings. Second, radiologists often do not describe VCFs if they remain stable over several years. Oncological patients undergo regular CT scans, often quarterly, over multiple years, whereas the current analysis is performed at a single time point. It is therefore likely that some fractures detected by the software during the study were previously reported in an earlier scan, with no significant changes in the current examination, leading to their omission in the radiology report. Finally, the DL software is highly sensitive compared to the radiologists’ eye in detecting grade 1 VCFs. However, these fractures have limited clinical or therapeutic relevance. Taken together, though the outputs of DL application for incidental VCF detection might provide useful clinical information, it becomes clinically relevant for patient outcomes only upon radiologist review.

Two clinical strategies of radiologist-DL tool interactions were previously proposed concerning incidental findings like VCF. On the one hand, DL-based applications can help incidental pathologies detection by acting as a second reader to catch missed cases. On the other hand, it can serve as a triage tool, prioritizing DL-flagged alerts, thus enhancing efficiency of incidental findings detection. However, this latter strategy risks to overlook false negative cases not flagged by DL-based application. It can be overcome by an adjustable balance between sensitivity and specificity of the application and clinical workflow optimization [55]. Regarding CINA-VCF Quantix, this application demonstrated to be a useful tool for complementary use in radiology practice, which would improve clinical workflow with the potential to reduce radiologists’ workload and improve vigilance for incidental VCF findings. We consider that both proposed strategies for DL tool implementation have the potential to be effective for the detection of VCFs in clinical routine. However, their efficacy should be evaluated individually, taking into account the specific needs of each clinical center, its area of specialization, and the clinical protocols in place for management of patients with VCF findings.

This study had limitations. First, as a retrospective study, it did not assess the real-world clinical integration or the real-time impact on patient management. Additionally, the retrospective nature of the study may introduce selection bias. To balance this bias, all consecutive CT scans on a given period have been included. Second, it was a single-center study, limiting the generalizability of the results to other centers and medical practices. Moreover, the study focused exclusively on patients with oncological profiles, with specific characteristics related to age, treatment approaches, and follow-up. This population is probably over-exposed to osteoporosis and VCF, thus increasing these findings in the final cohort. Finally, although reported, we did not make use of the osteoporosis parameters (mean HU) provided by the application, as the primary aim of the study was to assess the diagnostic performance for VCF detection and the unreported rate of VCF-positive patients in routine oncological clinical practice within the oncology department.

## Conclusion

In summary, this study demonstrates the potential clinical benefit of the DL-based application integration into a clinical workflow in order to detect opportunistic VCFs on regular CT scans. Importantly, the application proved effective in detecting previously overlooked patients with severe (grade 3) VCFs in a tertiary cancer center, thereby facilitating their redirection to appropriate treatment pathways and offering the potential for improved clinical outcomes. The system can complement radiologists’ assessments, improving the identification of patients with VCFs for inclusion in prophylactic treatment pathways and reducing non-reported rates, therefore contributing to enhancing patient long-term outcomes.

## Supplementary Information

Below is the link to the electronic supplementary material.

Supplementary file1 (DOCX 1775 kb)
