# Supplementary material for: Deep learning-driven incidental detection of vertebral fractures in cancer patients: advancing diagnostic precision and clinical management
Source: Radiol Med. 2025 Aug 2;130(10):1669–79. doi: 10.1007/s11547-025-02058-z (PMC12546477; doi:10.1007/s11547-025-02058-z)

Supplementary figures

**Supplementary Figure 1. Particular cases in the flagged as positive by CINA (VCF Quantix) dataset (n=501). A.** Cementoplasty. Only 7% of patients had a history of cementoplasty. **B.** Osteoporosis. Almost 51 % of patients had osteoporosis. Mean HU of L1 trabecular attenuation of the presented case is 96 HU (physiological reference: 122-198 HU). **C.** Vertebral metastasis. Almost 32 % of patients had vertebral metastases.


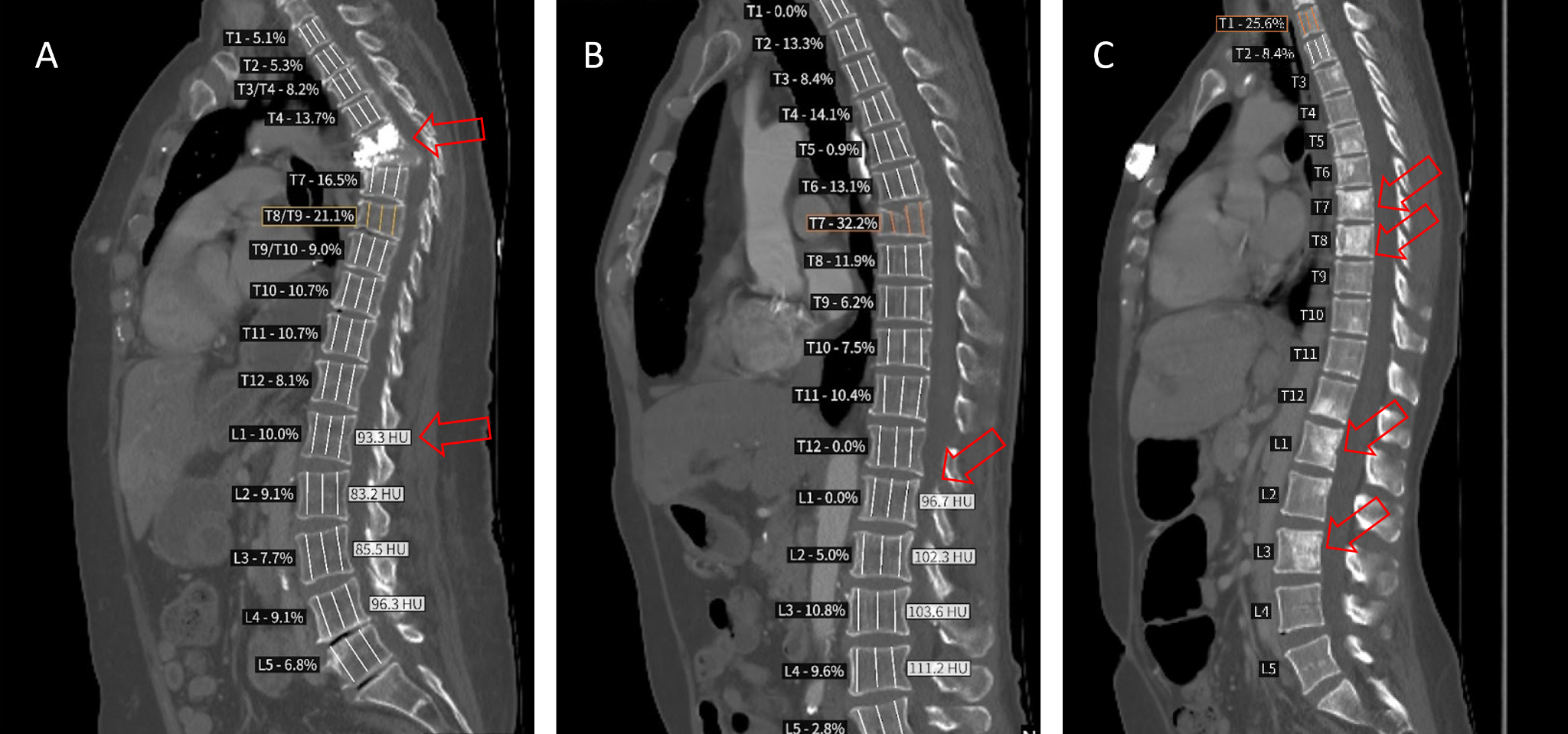


**Supplementary Figure 2. Cancer type distribution.** The patient distribution by cancer type is presented for all true positive cases (n=436).


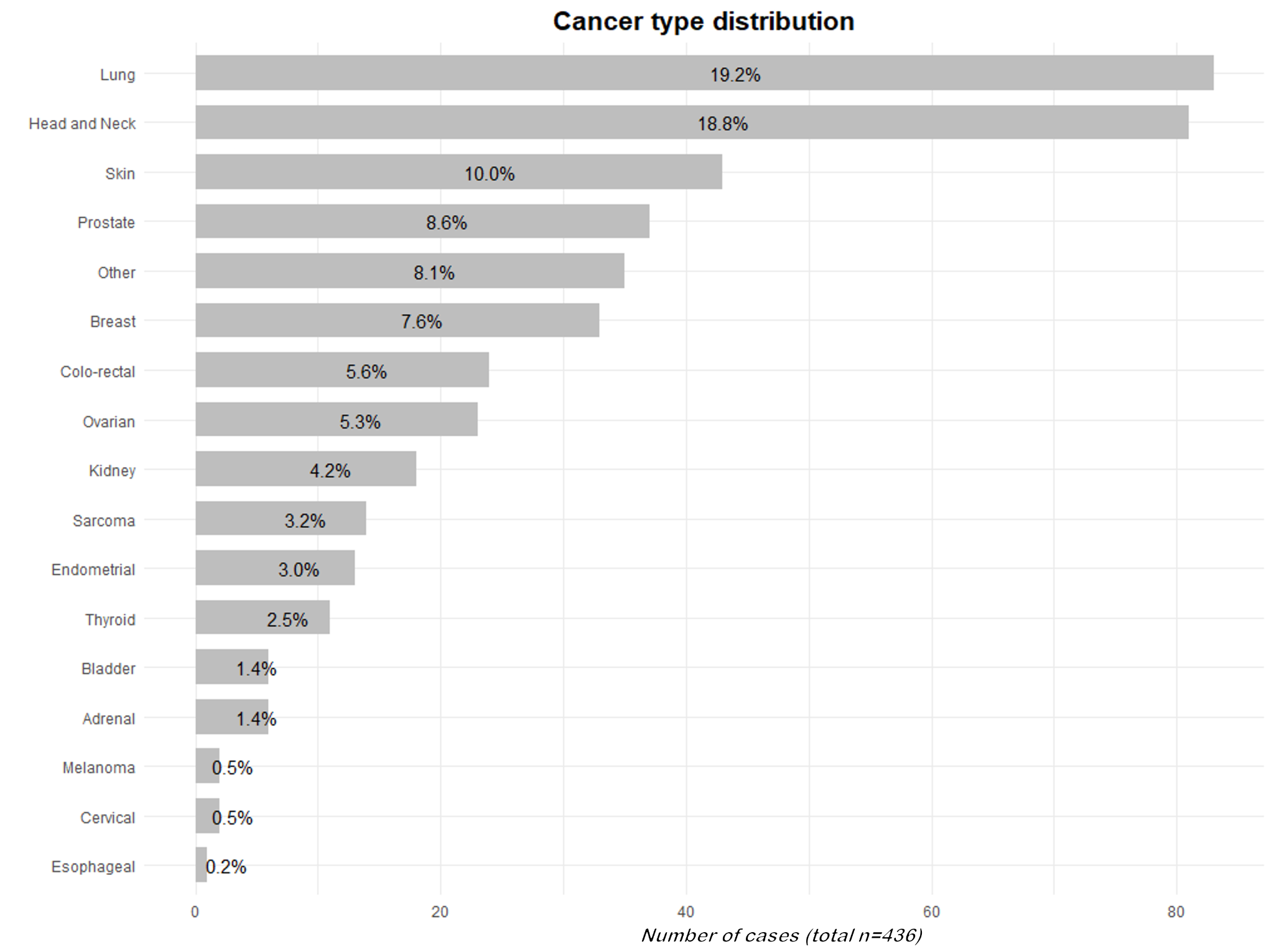

Supplement: Supplementary file 1 — Supplementary file1 (DOCX 1775 kb) [file 11547_2025_2058_MOESM1_ESM.docx]
